# Assessment of Cement Leakage in Decompressed Percutaneous Kyphoplasty

**DOI:** 10.3390/jcm13020345

**Published:** 2024-01-08

**Authors:** Shih-Hao Cheng, Wen-Hsiang Chou, Yu-Chuan Tsuei, William Chu, Woei-Chyn Chu

**Affiliations:** 1 Institute of Biomedical Engineering, National Yang-Ming Chiao-Tung University, Taipei 11221, Taiwan; franchpaladin.y@nycu.edu.tw (S.-H.C.); dontspider@yahoo.com.tw (Y.-C.T.); williamchu63214789@gmail.com (W.C.); 2 Department of Orthopedics, Cheng Hsin General Hospital, Taipei 11221, Taiwan; ch6215@chgh.org.tw; 3 School of Nursing, National Taipei University of Nursing and Health Sciences, Taipei 11221, Taiwan

**Keywords:** cement leakage, high-viscosity cement, kyphoplasty, osteoporotic compression fracture, vertebroplasty

## Abstract

Symptomatic osteoporotic compression fractures are commonly addressed through vertebroplasty and kyphoplasty. However, cement leakage poses a significant risk of neurological damage. We introduced “aspiration percutaneous kyphoplasty”, also known as “decompressed kyphoplasty”, as a method to mitigate cement leakage and conducted a comparative analysis with high viscosity cement vertebroplasty. We conducted a retrospective study that included 136 patients with single-level osteoporotic compression fractures. Among them, 70 patients underwent high viscosity cement vertebroplasty, while 66 patients received decompressed percutaneous kyphoplasty with low-viscosity cement. Comparison parameters included cement leakage rates, kyphotic angle alterations, and the occurrence of adjacent segment fractures. The overall cement leakage rate favored the decompressed kyphoplasty group (9.1% vs. 18.6%), although statistical significance was not achieved (*p* = 0.111). Nonetheless, the risk of intradiscal leakage significantly reduced in the decompressed kyphoplasty cohort (*p* = 0.011), which was particularly evident in cases lacking the preoperative cleft sign on X-rays. Kyphotic angle changes and the risk of adjacent segment collapse exhibited similar outcomes (*p* = 0.739 and 0.522, respectively). We concluded that decompressed kyphoplasty demonstrates efficacy in reducing intradiscal cement leakage, particularly benefiting patients without the preoperative cleft sign on X-rays by preventing intradiscal leakage.

## 1. Introduction

Osteoporotic compression fractures are a common occurrence in the geriatric population, leading to disability and great social burden [1,2,3]. Injuries of this nature often present a spectrum of symptoms, ranging from mild back soreness to debilitating pain that significantly impacts daily activities. Conservative treatments encompass a range from bed rest to osteoporotic medications to bracing. While many compression fractures respond well to conservative management, severe cases may necessitate surgical intervention [4,5]. Percutaneous vertebroplasty and kyphoplasty have been used to treat symptomatic osteoporotic compression fractures worldwide and have demonstrated satisfactory results [6,7,8,9]. Some patients experience immediate pain relief after the procedure. Although the procedure provides good clinical outcomes, the risk of cement leakage is constantly an important concern [10,11]. Studies have reported that the risk of cement leakage is underestimated and was higher than we have observed on radiographic examination. When using a CT scan instead of a routine X ray for leakage detection, the cement leakage rate may be as high as 81% [10]. Despite the often asymptomatic nature of most leakages, certain cases can lead to severe complications such as neurological injury or pulmonary embolism [12], potentially resulting in permanent disability or even mortality. Therefore, surgeons put great effort into identifying risk factors and reducing the occurrence of cement leakage. In earlier research, factors including fracture patterns, surgical technique, injury duration, and cement viscosity were identified to be factors affecting cement leakage [13,14,15]. Moreover, a range of surgical techniques and devices has emerged to mitigate leakage risks [16]. Notably, vertebroplasty employing high-viscosity cement has gained traction as an increasingly utilized solution for managing osteoporotic compression fractures. This type of cement has a shorter liquid phase and was claimed to be more predictable during injection [17,18,19,20]. Lower fluidity of the cement leads to slower cement diffusion in cancellous bone. The surgeon has enough reaction time to discontinue cement installation immediately whenever the cement is close to the cortical bone defect near the fracture site, thus reducing the risk of cement leakage. However, the effectiveness of high-viscosity cement is not well understood, and there is a lack of data regarding long-term outcomes. The distribution of the cement inside the vertebral body is of additional concern. The cement cannot penetrate into the cancellous bone and medullary canal as effectively as the cement of lower viscosity [21,22]. The uneven distribution and filling of the cement may lead to poorer biomechanical strength and may increase the subsequent adjacent segment collapse [23,24,25,26]. There are few studies reporting the kyphotic angle change and adjacent segment fractures in the literature review.

In the previous study, we described how a differential pressure technique was used as a driving force to guide “low viscosity” bone cement through the vertebral fracture site. We created a negative pressure environment inside the vertebral body by connecting a suction pump to one trocar. The flow and distribution of the injected cement between the two trocar tips can be “guided”. Our findings demonstrated that bone cement leakage can be significantly reduced [27]. However, the long-term outcomes of this procedure were never compared to high-viscosity cement vertebroplasty. In this study, we aimed to compare the risk of the cement leakage rate, radiographic outcome and rate of adjacent segment fracture between vertebroplasty with high-viscosity cement and decompressed kyphoplasty with low-viscosity cement.

## 2. Materials and Methods

### 2.1. Study Design

We retrospectively reviewed the patients who received vertebroplasty or kyphoplasty in our hospital from 1 March 2017 to 1 March 2019. Pre-operative and post-operative imaging, charts and surgery reports were reviewed. All of the surgeries were performed by the same surgeon with over 20 years’ experience in spinal surgery. 

### 2.2. Patient Inclusion Criteria

Each patient included in the study had a single-level osteoporotic compression fracture and received either decompressed kyphoplasty with low-viscosity cement (Simplex Bone Cement Radiopaque, Howmedica, Mahwah, NJ, USA) or vertebroplasty with high-viscosity cement (Confidence, Johnson & Johnson, New Brunswick, NJ, USA). All patients were diagnosed with osteoporotic compression fractures based on a history of minor trauma, clinical symptoms, plain-film X-rays, and magnetic resonance imaging (MRI) prior to surgery. There must have been at least four weeks of conservative treatment with bed rest, adequate analgesic medication, and bracing, before surgery. All patients were followed up more than 12 months after the surgery.

### 2.3. Exclusion Criteria

Patients who suffered from high-energy traumatic compression fractures, pathologic compression fractures caused by tumor or infection, old compression fractures more than three months that have healed and burst fractures were excluded. Patients who had multiple-level procedures were precluded from analysis as well. Patients with previous compression fractures or spinal instrumentation over the adjacent level were also excluded, as these factors might affect the risk of an adjacent compression fracture. Patients who did not have radiographic follow-up for more than 12 months were also excluded.

### 2.4. Surgical Technique

#### 2.4.1. Decompressed Kyphoplasty

We used the decompressed technique as reported in a previous article to guide the direction of the cement flow [27]. All procedures were performed under general anesthesia or intravenous sedation anesthesia. The patient was placed in a prone position on a radiolucent table, adequately padded to ensure stability and a slight lordotic posture. Using C-arm guidance, we located the bilateral pedicles at the fractured level. Two 8-gauge needles were introduced through bilateral pedicles into the vertebral body. The kyphoplasty balloon (Medtronic, Minneapolis, MN, USA) was inserted into the collapsed body and inflated from each trocar in order to reduce the collapsed body height. Once the pressure reached 200 Psi or the vertebral body height was restored, the balloon was deflated and removed. The vertebral body height of the above and below level was used as a reference of reduction target for the injury level. We connected an on-wall suction system within the operating room, set at 600 mmHg, to one trocar (acting as a venting portal) to establish a negative pressure environment within the fractured site. Then, we flushed the system with normal saline from the contralateral trocar (injection portal) to remove the debris inside the trocar and confirmed the patency of cement pathway. 

**Figure 1:**
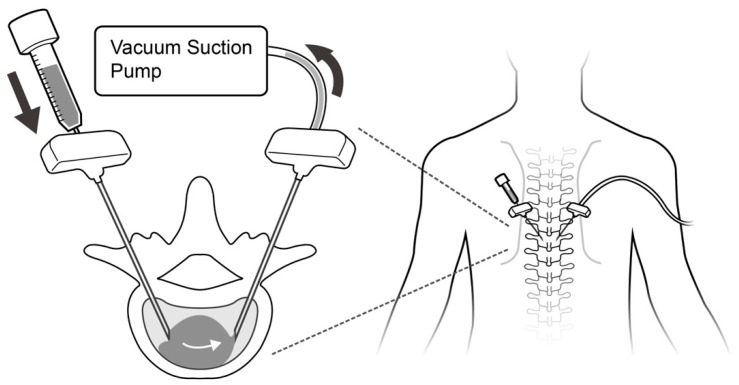
Schematic diagram of the decompressed kyphoplasty procedure. The venting portal was connected to a suction pump, while the injection portal was connected to a syringe for cement injection. The arrows represented the direction of cement flow in the pathway.

**Figure 2:**
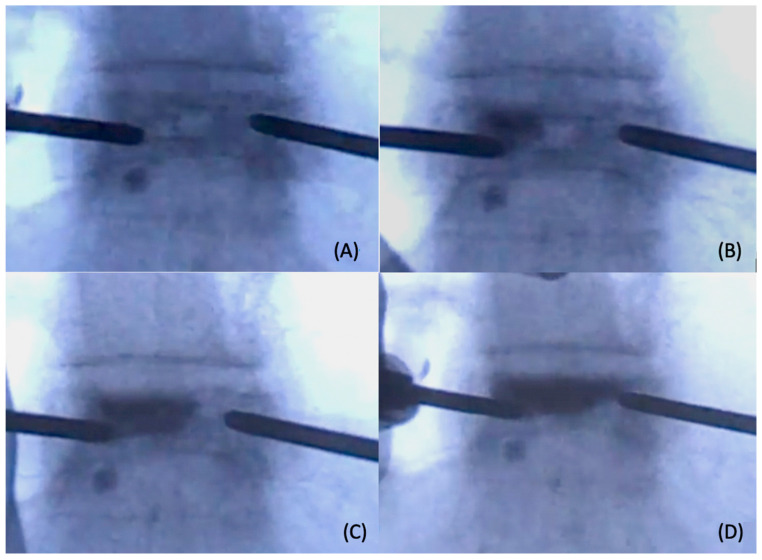
A series of real-time fluoroscopic images taken during cement injection in decompressed kyphoplasty. The cement infused gradually from the injection portal to the venting portal under the guidance of pressure gradience (**A**–**D**).

We utilized Simplex Bone Cement Radiopaque (Howmedica, NJ, USA), a low-temperature, low-viscosity and radiopaque bone cement, in all decompressed kyphoplasty procedures conducted on our patients. The low-viscosity cement was prepared within an operating room environment maintained at a temperature ranging approximately between 18 °C and 20 °C. The bone cement powder and liquid components underwent a thorough mixing process for forty-five seconds before being loaded into a 3cc syringe. Two minutes following the mixing, the bone cement was manually injected from the contralateral side using a 3cc syringe without forced injection (Figure 1). The cement distribution can be observed under serial fluoroscopy images (Figure 2). Some cement may come out from the venting portal but can be ignored as long as the suction tube remains in place and the negative pressure environment inside vertebral body was not compromised. Once the cement fully occupied the fracture site, or cement no longer emerged from the venting portal anymore, the installation of cement was stopped, and the trocars were removed after cement hardening. The whole procedure was performed under fluoroscopic guidance. The details of the technique were provided in our previous work [27,28].

#### 2.4.2. Vertebroplasty with High Viscosity Cement

The positioning of the patient, anesthesia methods and surgical approach were the same for both groups, except that 13-gauge trocars were inserted through bilateral pedicles instead. Each patient received the same high-viscosity cement (Confidence, Johnson & Johnson, NJ, USA). The cement was prepared using the manufacturer-provided mixer for 45 s and subsequently loaded into the delivery system for 2 min at room temperature, following a process similar to the kyphoplasty procedure. High-viscosity cement was then injected from trocars using the hydraulic delivery system that came with the package. The endpoint of the cement injection was achieved with adequate cement filling of the fracture site under fluoroscopy in both anterior–posterior and lateral views. The procedure was also stopped when cement leakage occurred.

#### 2.4.3. Post-Operative Care

All patients were required to wear a back brace for three months following surgery. Post-operative bone mineral density was routinely measured using dual-energy X-ray absorptiometry (DXA). Anti-osteoporotic treatments, such as exercise, nutritional supplements and pharmacologic treatments, were prescribed based on the T score and fracture risk. The anti-osteoporotic agents were chosen in accordance with the clinical practice guidelines of the American Association of Clinical Endocrinologists/American College of Endocrinology.

### 2.5. Radiographic Results

**Figure 3:**
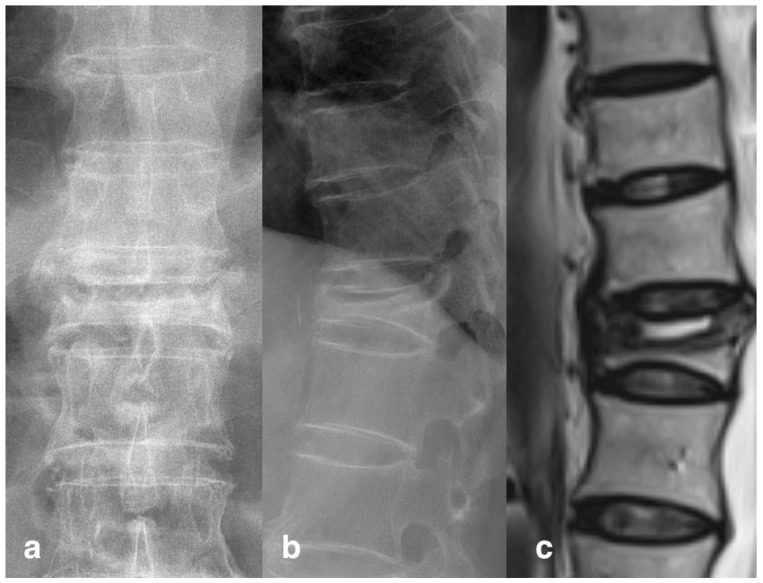
Vertebral cleft sign. The radiography of a patient with T12 compression fracture showed a typical cleft sign in (**a**) AP view and (**b**) lateral view. The sagittal view on T2 weight imaging MRI (**c**) also revealed a fluid-containing empty space in vertebral body.

Pre-operative and post-operative kyphotic angle and cement leakage were evaluated using X-rays. The kyphotic angle was defined as the angle between the extension of the upper and lower endplates, which was measured independently by two authors; an average value was then calculated. Presence of cement leakage, leakage type and occurrence of an adjacent segment compression fracture were also determined by the same two authors. Cement leakage was further divided into four groups according to location, including paravertebral, intradiscal, epidural and intra-venous types. The cleft sign, or vacuum phenomenon (Figure 3), was defined as a radiolucent linear or semilunar shadow on either the frontal or lateral X-ray [29]. All the X rays were independently reviewed by the two authors without knowing the name or procedure that the patients had received, and they made the initial judgment. If the results were inconsistent, the third surgeon with over 20 years’ experience was consulted to reach an agreement. Among all patients, there were six patients’ X-rays that required further consultation, but a consensus was reached for all of them after discussion.

### 2.6. Statistics Analysis

The data were processed with SPSS 22 (IBM Corporation, Armonk, NY, USA). The general patient information was presented using descriptive statistics. Category variables, such as gender, presentation of cement leakage and adjacent segment collapse, were compared using the Chi-square test. We compared the continued variables, including age, kyphotic angle changes and follow-up duration between the two groups, using an independent sample *t*-test. The kyphotic angle difference before and after operation was compared with a dependent sample t-test. Subgroup analysis was performed in the group receiving the high-viscosity cement, in order to determine the effect of the vacuum phenomenon. Testing for normality was performed using the built-in function of SPSS software. The abovementioned data were assessed visually with a normal Q-Q plot and numerically with a Shapiro–Wilk test of normality. A *p*-value less than 0.05 was considered statistically significant.

## 3. Results

### 3.1. Patient Demographics

A total of 136 patients, comprising 23 males and 113 females, were included in our study. In total, 70 patients received vertebroplasty with high-viscosity cement (Group 1) and 66 patients received decompressed kyphoplasty with low-viscosity cement (Group 2). There was no significant difference in age, sex or follow-up duration between the two groups. Most of the operated levels are between T11 to L2 (Table 1).

### 3.2. Radiography Results

**Figure 4:**
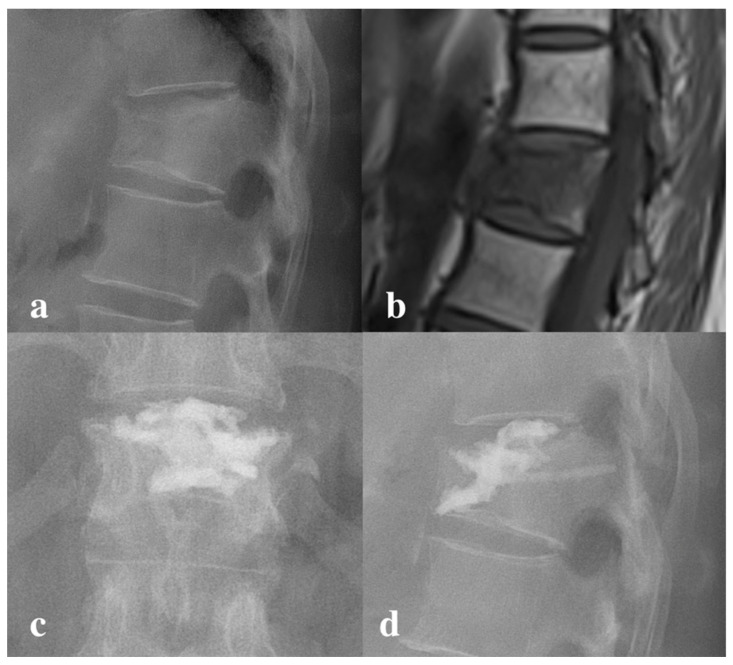
Intradiscal cement leakage. A 61 year-old female received vertebroplasty with high-viscosity cement because of the compression fracture of T12. There are no cleft signs on preoperative (**a**) X-ray and (**b**) MRI T1 weight image. Postoperative X-ray showed intradiscal cement leakage in both (**c**) AP view and (**d**) lateral view.

Both groups showed a significant improvement in the kyphotic angle after the surgery (*p* < 0.001). On the contrary, the kyphotic angle before and after surgery was similar between the two groups (*p* = 0.551, *p* = 0.651, respectively). The kyphotic angle changes were not significant (*p* = 0.739). There were 13 patients in group 1 and 6 patients in group 2 that demonstrated radiographic leakage. The overall leakage rate did not reach a significant level for the two groups (*p* = 0.111). However, there were nine patients in group 1 that showed intradiscal leakage (Figure 4), which is significantly higher than the single patient in group 2 (*p* = 0.011). Of note, all intradiscal leakage occurred in patients whose preoperative X-ray did not show a cleft sign, which is significant in the subgroup analysis (*p* = 0.002). There was no epidural leakage or intra-venous leakage in either group (Table 2).

### 3.3. Complications

There were no serious complications, e.g., symptomatic pulmonary embolism, neurologic symptoms or infection, in either group. During follow-up, 18 adjacent segment fractures (13.2%) occurred, mostly at the immediately superior level. However, there was no difference between the two groups, despite the higher intradiscal leakage rate in group 1.

## 4. Discussion

Our study reported that using decompressed kyphoplasty resulted in a lower rate of intradiscal cement leakage, but it did not lead to a lower adjacent segment fracture rate. Intradiscal cement leakage is highly associated with the absence of a radiographic cleft sign when using high viscosity cement. 

Both vertebroplasty and kyphoplasty have been used to treat osteoporotic compression fractures for decades, with good results [30]. During the performance of the procedure, cement leakage is a common complication and can cause devastating consequences. According to previous studies, the leakage rate is heavily underestimated and may be higher than 70% [10]. Preventing cement leakage has always been an important issue. Recently, high-viscosity cement was applied to treat compression fractures, in an attempt to reduce the cement leakage rate [18,31]. In this present work, the leakage rate using high-viscosity cement is 18.6%, which is lower than previous reports regarding traditional vertebroplasty. However, we observed a significantly higher intradiscal leakage rate in the high-viscosity cement group as compared to the decompressed kyphoplasty technique [27]. In our prior animal investigation, we noted the efficacy of decompressed techniques in minimizing cement leakage in a goat model [28]. This phenomenon can be explained by the different cement driving forces of the two techniques. Cement in decompressed kyphoplasty follows the guide of the pressure gradient created by a suction force [27]. Once the balloon is inflated and creates a cavity in the vertebral body, it forms a corridor for the cement to reside in. After applying negative pressure from one trocar, cement installed from another trocar was drawn to the contralateral side passively under the guidance of the negative pressure. Conversely, the distribution of the high-viscosity cement is mainly dependent on gravity and active pressure provided manually by the surgeon [28]. The cement often diffused profoundly from the tip of the trocar and aggregated at the site with the lowest resistance [32]. When the endplate defect existed, the cement can easily flow into the relative empty disc space [33]. This phenomenon might be more evident with the high-viscosity cement, which has a poorer ability to penetrate into cancellous bone [34], causing a higher risk of intradiscal leakage. Uneven cement distribution may be linked to suboptimal cement integration, potentially resulting in cement displacement following the surgical procedure [35].

We also discovered an interesting finding in the subgroup analysis, in that the intradiscal leakage is highly correlated to whether the “cleft sign” is observed on the X-ray. The risk of intradiscal leakage is higher when the pre-operative radiography does not have a “cleft sign” visible (*p* = 0.02). This “cleft sign” or “vacuum phenomenon” represented a pre-existing space inside the vertebral body, either occupied by gas or fluid, indicating a non-healing vertebral collapse or pseudoarthrosis [36]. This space could be enlarged when the patient was put into a prone position and postural reduction was performed, forming an ideal space for cement to fill [37]. The risk of intradiscal leakage is lower when this space is presented. We speculate that it is because the cement can accumulate in the space inside the vertebral body, instead of being infiltrated randomly out of fracture fissures. In our study, all nine patients with intradiscal leakage occurred on the fractures which did not demonstrate a pre-existing vacuum phenomenon, with a concomitant upper endplate fracture. This suggests that patients without the vacuum phenomenon on their pre-operative X-ray are at a higher risk of intradiscal disk leakage. Historically, the relationship between the cleft sign and cement leakage in vertebroplasty had been widely discussed. Some studies indicated that a cleft sign did not affect the risk of cement leakage but rather influenced the leakage pattern [38,39]. Other studies revealed a significantly lower cement leakage rate in conjunction with the presence of a cleft sign [40,41]. There were also papers which demonstrated the opposite results, where the cleft sign may be a risk factor for increased cement leakage [42]. Currently, the meta-analysis of Zhan et al. reviewed the evidence regarding cleft signs and concluded that the phenomenon is a risk factor for cement leakage [43]. This result is contrary to our findings for high-viscosity cement vertebroplasty. Since the technique for vertebroplasty is similar, we presumed that unique physical properties of high-viscosity cement may lead to a different influence of a cleft sign with vertebroplasty.

Currently, the long-term effect of intradiscal cement leakage is still not well known. Some authors have suggested cement injection into the intervertebral disc to treat discogenic degeneration-related back pain and radiculopathy without long term negative effects [44]. However, this study was designed to treat lumbar disc degeneration patients, which is completely different from osteoporotic compression patients, and should be discussed separately. In treating compression fractures, unintended, uncontrolled intradiscal cement leak should be avoided. While this type of cement leakage does not result in neurologic injury, it raises concerns about adjacent segment fractures due to uneven pressure distribution [45]. While some studies reported that the leakage did not induce adjacent vertebral body collapse [46,47], others obtained opposite results [48]. In addition to osteoporosis, excessive balloon distraction during surgery and multiple levels surgery, intradiscal leakage could be an independent risk factor for adjacent fractures. In the meantime, many studies revealed that bone cement may be toxic to the intervertebral disc and that it accelerates degeneration [49,50,51]. In our study, despite the higher intradiscal leakage rate, we did not observe a higher rate of adjacent segment fractures in the high-viscosity cement group. However, considering bone cement’s potential damage to disc tissue, we should put all efforts into preventing this complication, including the selection of an appropriate technique for patients without a cleft sign on pre-operative X-rays.

During vertebroplasty and kyphoplasty procedures, the preparation of cement stands as a crucial step influencing the physical characteristics of the bone cement. Past research has highlighted several contributing factors, including temperature, humidity, solution ratio, blending speed during preparation, and the introduction of blood or normal saline [52,53]. In our established protocol, we ensured precise cement preparation within a controlled-temperature environment in the operating room to mitigate temperature-related effects. Employing a low-viscosity cement, we initiated the injection process two minutes after blending to maintain the desired fluidity. It is crucial to avoid excessively sticky cement, as it may adhere to the trocha, impeding the formation of negative pressure and rendering high-viscosity cement unsuitable for decompressed kyphoplasty. Before cement injection, we connected a suction device to the trocar and flushed it with normal saline to eliminate blood, debris and residual fluids from the fracture site. This procedural step minimizes the impact of these materials on the bone cement’s physical properties, facilitating improved bonding between the trabecular bone and the cement [54].

The study was limited by its retrospective nature, and a well-designed prospective randomized controlled trial is necessary in order to confirm our conclusions. The patients were evaluated with plain-film radiography following their surgery, which may have under-estimated the leakage rate. For adjacent segment fracture, the patients only underwent a 12-month follow-up. Although previous studies reported that adjacent segment fracture following vertebroplasty or kyphoplasty often happened within three months of the procedure [55,56,57], a longer follow-up period may be required in order to confirm the long-term effect of the different bone cements. No significant complications were noted in either patient group, which might be linked to the limited participant pool. However, it is crucial to acknowledge the potential occurrence of rare complications, such as cement implantation syndrome, as discussed in a prior study [58]. Conducting a more extensive study with a larger participant cohort is essential to substantiate the effect of decompressed kyphoplasty on these complications.

## 5. Conclusions

When compared to vertebroplasty with high-viscosity cement, decompressed kyphoplasty with low-viscosity cement can reduce cement leakage, particularly intradiscal cement leakage. On X-ray, the absence of the cleft sign is a strong predictor of intradiscal cement leakage. Surgeons might find the decompressed kyphoplasty technique beneficial in reducing complications in these scenarios. However, further prospective studies involving a larger sample size are warranted to comprehensively assess the effectiveness of decompressed kyphoplasty in mitigating other rare complications.

## Figures and Tables

**Table 1:** Demographic of patients.

		Group 1 (*n* = 70) High-Viscosity Cement	Group 2 (*n* = 66) Decompressed Kyphoplasty	*p* Value
Age (Mean ± SD)		76.46 ± 8.38	78.52 ± 7.18	0.120
Sex (male/female)		8/62	15/51	0.109
Follow up (days) (Mean ± SD)		524.7 ± 96.5	531.9 ± 94.6	0.656
Injured level (*n*/%)	Above T11	2 (2.8%)	3 (4.5%)	0.854
	T11	16 (22.9%)	14 (21.2%)	
	T12	24 (34.3%)	22 (33.3%)	
	L1	17 (24.3%)	12 (18.2%)	
	L2	8 (11.4%)	12 (18.2%)	
	Below L2	3 (4.3%)	3 (4.5%)	
T score (Mean ± SD)		−3.19 ± 0.50	−3.24 ± 0.54	0.612
Operation time (minutes) (Mean ± SD)		29.82 ± 7.71	40.21 ± 12.51	<0.001

**Table 2:** Radiographic results.

		Group 1 (*n* = 70) High-Viscosity Cement	Group 2 (*n* = 66) Decompressed Kyphoplasty	*p* Value
Kyphotic angle (degree) (Mean ± SD)	Pre-operation	21.47 ± 6.86	20.35 ± 8.64	0.551
	Post-operation	15.19 ± 5.74	14.43 ± 7.91	0.651
	Angle change	6.28 ± 4.32	5.91 ± 4.80	0.739
Cleft sign positive (*n*/%)		20 (28.6%)	24 (36.4%)	0.332
Adjacent segment fractures (*n*/%)	Above level	7 (10%)	8(12.1%)	0.693
	Below level	1(1.4%)	2(3.0%)	0.525
	Total	8(11.4%)	10(15.2%)	0.522
Adjacent fracture time(days)		24.0(10–42)	21.80(12–37)	0.762
Cement leakage type (*n*/%)	Paravertebral	4 (5.7%)	5 (7.6%)	0.663
	Epidural	0	0	
	Intradiscal	9 (12.9%)	1 (1.5%)	0.011
	Intra-venous	0	0	
	total	13 (18.6%)	6 (9.1%)	0.111

## Data Availability

The data used to support the finding of this study are available from the corresponding author upon request.
